# Case for diagnosis. Erythematous and pruritic papules on forearms^☆^^☆☆^

**DOI:** 10.1016/j.abd.2019.06.006

**Published:** 2020-02-12

**Authors:** Aline Palitot Santana, Alberto Eduardo Cox Cardoso, Rafaela Tenório Passos, Íris Sampaio Costa Ferreira

**Affiliations:** Dermatology Clinic, Hospital Universitário Professor Alberto Antunes, Universidade Federal de Alagoas, Maceió, AL, Brazil

**Keywords:** Ectoparasitic infestations, Mite infestations, Zoonoses

## Abstract

Gamasoidosis is a poorly known and underdiagnosed mite infestation. It is characterized by the presence of erythematous and flattened papules that are quite pruritic, and can affect any region of the body, with preference for areas of folds. This article reports a case of the disease caused by mites of the species *Dermanyssus gallinae*. Increasingly, the agents that cause this disease are found in urban environments, increasing the incidence of people affected by the disease. This dermatosis has a self-limiting clinical picture and the treatment is done with the use of topical corticosteroids and oral antihistamines.

## Case report

**Figure 1:**
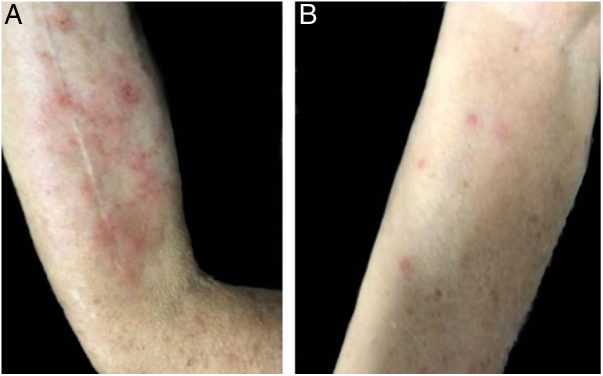
Erythematous papules with pruritus and excoriation in right arm (A) and forearm (B).

**Figure 2:**
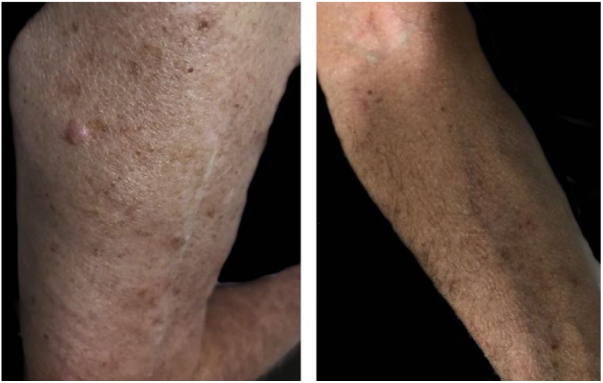
Remission of lesions in right arm (A) and forearm (B).

**Figure 3:**
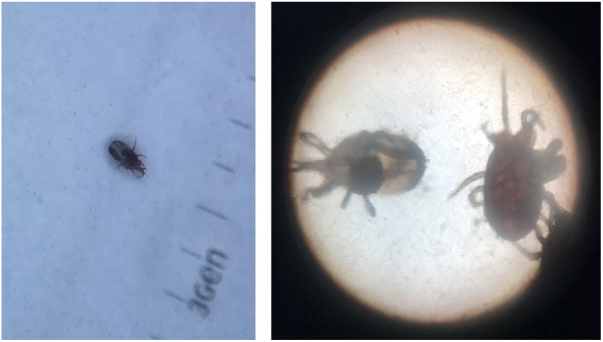
(A) Dermatoscopy of the mite. (B) Identification of mites of the species *Dermanyssus gallinae* by optical microscopy.

A 77 year-old male patient sought dermatological care due to extremely pruritic body lesions that began about 15 days ago. He denied comorbidities. Dermatological examination showed erythematous-papular lesions in the body, mainly in the upper limbs (Fig. 1). Oral antihistamine, topical clobetasol propionate and emollient have been prescribed. The patient reported having found poultry mite in his room, and, after being alerted, searched and found the mites in a nest of birds inside the air-conditioning box. After the use of prescribed medications, the patient evolved with improvement of the symptoms (Fig. 2), and in the subsequent consultation he brought some specimens of the mites found in his room, identified as being of the species *Dermanyssus gallinae* (Fig. 3).

## Discussion

Gamasoidosis or avian-mite dermatites is a little known and underdiagnosed infestation that is becoming increasingly common, especially in urban environment with the proliferation of pigeons in cities from nests built on roofs, windows and air-conditioning boxes. There are also nosocomial cases.^1^, ^2^

It is mainly caused by mites of the species *D. gallinae*, but other species may be involved, such as *Ornithonyssus sylviarum*, *Ornithonyssus bursa* and *Dermanyssus avium*.^1^, ^2^ They are popularly known as “poultry mite”, “roost mite” and “chicken mite”.^3^ These arthropods, which measure about 1 mm in diameter, are temporary hematophagous ectoparasites of domestic and wild birds, mainly infesting chickens, turkeys, pigeons and birds. They can also feed on other species, including humans, being found in hosts only when they are feeding at night. The rest of its biological cycle is carried out outside the host, colonizing nests, cracks and grooves, which become its hiding place. Infected birds, in addition to skin lesions, may present severe neurological conditions but in humans symptoms are exclusively cutaneous.^2^

Lesions usually resemble those of scabies and pediculosis. They are commonly flattened erythematous papules, very pruritic, and can affect any part of the body.^2^, ^4^ No dermatoscopic criteria have been described for the disease, but dermatoscope may help to exclude delusional parasitosis diagnosis.^1^

Dermatitis caused by parasitic arthropods of birds is often overlooked even neglected, but must always be remembered in cases of acute prurigo.^3^ Clinical condition is self-limiting and usually regresses spontaneously, but symptomatic treatment with topical corticosteroids and oral antihistamines can be done. Prevention of new cases is done with strict surveillance, removal of nests of the birds containing mites, and with cleaning and disinfestation of affected area with acaricide.^2^, ^5^, ^6^

## Financial support

None declared.

## Authors’ contributions

Aline Palitot Santana: Conception and planning of the study; elaboration and writing of the manuscript; critical review of the manuscript.

Alberto Eduardo Cox Cardoso: Approval of the final version of the manuscript; critical review of the literature.

Rafaela Tenório Passos: Elaboration and writing of the manuscript; critical review of the manuscript.

Íris Sampaio Costa Ferreira: Approval of the final version of the manuscript; critical review of the manuscript.

## Conflicts of interest

None declared.
